# Assessing pathogenicity of *MLH1 *variants by co-expression of human *MLH1 *and *PMS2 *genes in yeast

**DOI:** 10.1186/1471-2407-9-382

**Published:** 2009-10-28

**Authors:** Matjaz Vogelsang, Aleksandra Comino, Neja Zupanec, Petra Hudler, Radovan Komel

**Affiliations:** 1 Department for Biosynthesis and Biotransformation, National Institute of Chemistry, Hajdrihova 19, SI-1001 Ljubljana, Slovenia; 2 Medical Center for Molecular Biology, Faculty of Medicine, University of Ljubljana, Vrazov trg 2, SI-1000 Ljubljana, Slovenia

## Abstract

**Background:**

Loss of DNA mismatch repair (MMR) in humans, mainly due to mutations in the *hMLH1 *gene, is linked to hereditary nonpolyposis colorectal cancer (HNPCC). Because not all *MLH1 *alterations result in loss of MMR function, accurate characterization of variants and their classification in terms of their effect on MMR function is essential for reliable genetic testing and effective treatment. To date, *in vivo *assays for functional characterization of *MLH1 *mutations performed in various model systems have used episomal expression of the modified MMR genes. We describe here a novel approach to determine accurately the functional significance of *hMLH1 *mutations *in vivo*, based on co-expression of human MLH1 and PMS2 in yeast cells.

**Methods:**

Yeast *MLH1 *and *PMS1 *genes, whose protein products form the MutLα complex, were replaced by human orthologs directly on yeast chromosomes by homologous recombination, and the resulting MMR activity was tested.

**Results:**

The yeast strain co-expressing hMLH1 and hPMS2 exhibited the same mutation rate as the wild-type. Eight cancer-related *MLH1 *variants were introduced, using the same approach, into the prepared yeast model, and their effect on MMR function was determined. Five variants (A92P, S93G, I219V, K618R and K618T) were classified as non-pathogenic, whereas variants T117M, Y646C and R659Q were characterized as pathogenic.

**Conclusion:**

Results of our *in vivo *yeast-based approach correlate well with clinical data in five out of seven hMLH1 variants and the described model was thus shown to be useful for functional characterization of *MLH1 *variants in cancer patients found throughout the entire coding region of the gene.

## Background

Mismatch repair (MMR) genes are genome-stability genes. Mutations in these genes cause defects in the MMR pathway that can lead to carcinogenesis, as a result of accumulation of non-repaired post-replicational mis-incorporations in cancer contributing genes. Inherited germline mutations of MMR genes cause a predisposition to cancer, which can develop at an early age, as the next somatic mutation needs to affect only one, wild-type allele of a gene [1,2]. It has been established that many families with hereditary non-polyposis colorectal cancer (HNPCC) harbour potentially pathogenic mutations in MMR genes, particularly in *hMLH1 *and *hMSH2 *[3]. The disease manifests with high mortality rate if not detected and treated early, therefore presymptomatic genetic testing is required [4]. However, the functional effects of MMR mutations need to be characterized first. This is essential, not only for effective diagnosis of cancer predisposition, but also for choosing the appropriate chemotherapy, since it is known that MMR mutations can cause resistance to chemotherapeutic agents by insufficient induction of apoptosis [5].

There are over 450 germline allelic variants of *hMLH1 *and *hMSH2 *genes known to date and more are being found, but the functional relevance of each is very difficult to establish on clinical samples alone. Therefore many *in vitro *and *in vivo *assays have been developed for MMR gene mutation analysis, using various molecular genetics tools [6-16]. An *in vitro *functional analysis - involving Western immunoblotting - of four *hMLH1 *mutations found in HNPCC patients was described by Belvederesi et al. [6]. Several other strategies have been based on analysing the interaction between MMR variants, using the yeast two-hybrid system [7,8]. One recent study described the characterization of *MLH1 *variants in terms of multiple functional properties (e.g. protein expression/stability, nuclear localization, protein-protein interaction and MMR efficiency) [9]. *In vivo *assays have been performed in bacterial and yeast model systems, all of them using expression vectors with human MMR gene variants [10-13]. An alternative, commonly reported approach in yeast is the use of vectors with yeast MMR orthologs harbouring a mutation corresponding to those found in HNPCC patients, but the method is limited by the degree of homology between human and yeast genes [14,15]. *MLH1 *variants were also introduced into human cell lines to analyze their effects on apoptosis, proliferation, and regulation of mRNA expression, as well as expression of interacting proteins [16].

Although these studies have provided valuable clues to a better understanding of *MLH1 *variants, it is difficult to establish and compare their clinical relevance since a variety of strategies have been used. We believe that for reliable interpretation of functional effects of MLH1 gene mutations and their classification, all *MLH1 *mutations extending within the whole gene coding region should be analyzed using the same approach. In this study we are proposing a novel *in vivo *approach to determine the functional significance of *hMLH1 *mutations throughout the whole coding region of the gene. The *hPMS2 *and *hMLH1 *genes, whose proteins form the hMutLα complex, were each introduced into the yeast chromosome, replacing the yeast orthologs. Direct chromosomal integration enabled just one copy of a target gene to be introduced per cell. Both human orthologs were under control of the yeast promoter, and the original yeast genetic background was preserved. The functionality of mismatch repair was examined using a quantitative *in vivo *DNA MMR assay. To assess the functionality of the novel test system, nine different MLH1 amino acid replacements were introduced *in vivo *and evaluated for their pathogenicity. Results show that our *in vivo *yeast-based approach can help assess the pathogenic potential of cancer-related *hMLH1 *variants.

## Methods

### Yeast strain and vectors

The yeast Saccharomyces cerevisiae haploid strain used was W303-1A/RE966 (MATa ade2 can1-100 his3-11,15 leu2-3, 112 trp1-1 ura3-1) [17]. Cells were maintained in standard rich medium (YPD) [yeast extract/peptone/dextrose] and in selective synthetic medium (SD) [YNB: 0,67% w/v yeast nitrogen base, 2% w/v dextrose] supplemented with amino acids, depending on the auxotrophic requirements, and grown at 30°C. Media were solidified by addition of 2% w/v bacto-agar. The E. coli DH5α strain was used as bacterial host for plasmid propagation. Plasmid pCORE with KlURA3-kanMX4 counter-selectable reporter marker cassette (CORE) and plasmid pSH91 were provided by Francesca Storici [18] and Thomas D. Petes [19], respectively. The yeast expression vector pSH91 used for in vivo MMR assay contains an in-frame (GT)16.5 tract in the URA3 coding region. hMLH1 [GeneBank: NM000249] and hPMS2 [GeneBank: NM000535] cDNAs were obtained from pCMV-SPORT6 vector (ATCC, MGC-5172) and pSG-PMS2-wt, respectively. The latter plasmid was provided by Bert Vogelstein and Kenneth W. Kinzler [20]. Expression of yeast URA3 gene in pSH91 is under LEU2 gene promoter, so all test strains were transformed with Yep181 vector expressing LEU2 gene for restoration of leucine prototrophy [21].

### Construction of humanized yeast strains

**Figure 1:**
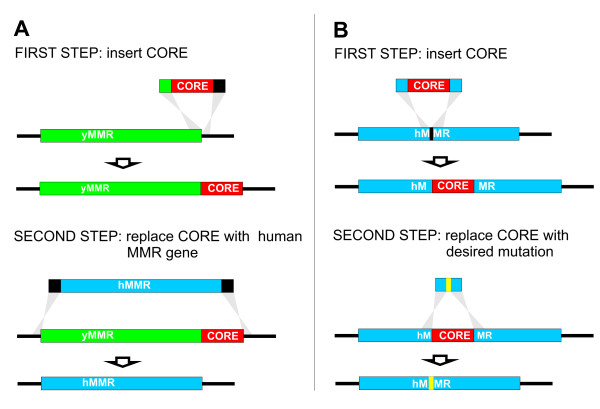
**Constructing humanized yeast strains with delitto perfetto system**. A. Replacing yeast MMR (*yMMR*) genes (i.e. yMLH1 and yPMS1) with their human orthologs (*hMMR*). First, the CORE cassette was introduced into yeast chromosome (*line*) after the stop codon of a yeast MMR gene, enabling the yeast gene to be still active during the second step of the process. Next, together with CORE cassette, the yeast gene was replaced with its human ortholog. B. Introducing missense alterations into *hMLH1 *gene in strain co-expressing *hMLH1 *and *hPMS2 *genes. First, the CORE cassette was introduced into *hMLH1*, replacing nucleotide of interest (*black vertical line*). Next, the CORE cassette was replaced by DNA fragment harbouring a single-nucleotide alteration (*yellow vertical line*). Genetic technique, *delitto perfetto*, is based on recombination event (*grey area*) between two identical strand of DNA.

A »*delitto perfetto*« method was used for constructing humanized yeast strains, as described by Storici et al. [18]. *Delitto Perfetto *is a two step approach for *in vivo *site-directed mutagenesis in yeast, using highly proficient homologous recombination system of *S cerevisiae*. The first step involves integration of a counterselectable (CORE) cassette in the region of interest. Subsequently, the CORE cassette is replaced with DNA fragment containing the mutation or the gene of interest (Figure 1). The CORE cassette allows the selection of yeast cells that receive the cassette during the first step, as well as selection of cells that lose the cassette in the second step of the process.

In this study, the counter-selectable reporter (CORE) cassette with *KlURA3 *and *kanMX4 *was amplified from pCORE and integrated into yeast chromosome XIII after the stop codon of the *yMLH1 *coding region (Figure 1A). 60- to 70-meric oligonucleotides (Sigma-Aldrich), consisting of 40- to 50-nucleotide flanking regions homologous with the appropriate yeast genomic target locus and a 20-nucleotide tract required for amplification from the vector were used for the amplification. DNA fragments were amplified by the polymerase chain reaction (PCR) using Platinum^® ^*Pfx *DNA polymerase (Invitrogen) and introduced into yeast cells with the LiAc transformation protocol [22]. Yeast clones that integrated CORE cassette into their genome were selected on YPD plates containing 200 μg/mL of geneticin (G418; Sigma-Aldrich). The CORE cassette was then completely removed by introducing complete *hMLH1 *cDNA into the yeast cells. Human *MLH1 *cDNA was previously amplified from pCMV-SPORT6 vector using 60-meric oligonucleotides. After transformation, the resulting Ura-cells, which lost CORE cassette, were isolated from synthetic complete medium containing 1 mg/L 5-fluororotic acid monohydrate (5-FOA; Toronto Research Chemicals Inc.). Isolated clones (strain MV200) were identified by PCR, using Taq polymerase (Eppendorf^® ^Master Mix), and the nucleotide sequences of the constructs were confirmed by DNA sequencing (Sequiserve).

To replace *yPMS1 *by *hPMS2 *directly on the chromosome (strain MV300), the CORE cassette was amplified with 60-meric oligonucleotides from pCORE and introduced after the stop codon of the yPMS1 gene in the yeast chromosome. The integrated CORE cassette, together with the yPMS1 gene, was then replaced by hPMS2 cDNA, previously amplified from vector pSG-PMS2-wt with oligonucleotides targeting yeast chromosome XIV. PCR products were introduced into the yeast cells and clones were isolated as described above. In order to obtain a yeast strain containing both *hMLH1 *and *hPMS2 *(strain MV400), the replacement of *yMLH1 *with *hMLH1 *was done in the strain MV300 already harbouring *hPMS2*.

The yeast *mlh1::kanMX *gene disruption was constructed by replacing the entire *yMLH1 *gene coding region with *kanMX *gene amplified from pFA6aKanMX4. Clones (strain MV100) were selected on YPD plates containing G418 and identified by PCR.

When constructing *MLH1 *single-nucleotide substitutions (Figure 1B), the CORE cassettes were amplified from pCORE with oligonucleotides targeting the human ortholog at the relevant codons. Integrative 3'-overlapping recombinant oligonucleotides harbouring the desired single-nucleotide substitutions were extended *in vitro *as described by Storici et al [18]. PCR products were again transformed into appropriate geneticin resistant yeast cells and clones were isolated as described above. All oligonucleotide sequences are available upon request.

### In vivo MMR assay and statistical analysis

The standardized *in vivo *MMR assay has been described in detail elsewhere [23]. The assay is based on stability of 33 bp GT tract, which is inserted in-frame the yeast *URA3 *gene in plasmid pSH91, as a measure of MMR efficiency. Instability of dinucleotide repeat is associated with DNA polymerase slipping during replication and alterations in tract length result in *ura3 *mutants, if not repaired by MMR. Mutant *ura3 *cells can be measured by their resistance to 5-FOA. Expression of the yeast *URA3 *gene in pSH91 is under *LEU2 *gene promoter, so all strains have to be leucine prototrophs.

In brief, plasmids pSH91 and Yep181 were first transformed [24] into the prepared yeast strains. At least 12 colonies of each analyzed yeast strain were picked from selective media plates (lacking tryptophan, leucine and uracil) and grown to saturation in SD supplemented with adenine and histidine to ensure that the starting cultures were homogeneous for URA3 cells with in-frame GT tracts. Cultures were diluted 1:1000 into SD supplemented with adenine, histidine and uracil, and grown to OD660 of 1. This allowed growth of all cells, including newly arising ura3 mutants. Each culture was then serially diluted and plated on SD plates supplemented with adenine, histidine and uracil, and the number of colonies was counted after a 3-day incubation to determine cell concentration. 100 μL aliquots of undiluted culture were plated on SD plates supplemented with adenine, histidine and uracil and containing 5-FOA. Colonies were counted after 3 days to calculate the concentration of ura3 mutants. Strains with increased instability of the GT tract, due to a disrupted MMR system, resulted in the ura3 mutant strain. Mutation rates were calculated using the method of the median [25]. For statistical analysis, Mann-Whitney tests were performed between all strains using GraphPad-InStat software. Differences in mutation rates were considered significant when P ≤ 0.05.

## Results

### Characterization of human MMR genes in yeast

**Figure 2:**
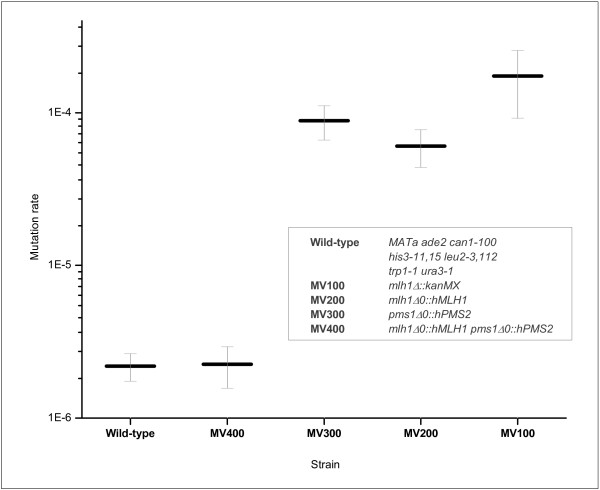
**The MMR efficiency in yeast strains harbouring endogenous and/or human MMR genes**. Each datum is the mean, with a 95% confidence interval indicated, of three independent experiments. Mean mutation rates are: Wild-type 2.19 × 10^-6 ^; MV100 1.74 × 10^4^; MV200 6.05 × 10^-5^; MV300 8.86 × 10^-5^; MV400 2.25 × 10^-6^. Genotypes of strains are boxed. All prepared strains originated from W303 background [17].

For functional analysis of HNPCC-related *hMLH1 *variants we prepared several humanized yeast strains with *hPMS2 *and different *hMLH1 *variants inserted into the genome and replacing their yeast orthologs. The function of the human MLH1 protein variants was determined with a standardized *in vivo *MMR assay measuring stability of the (GT)_16.5 _tract *in vivo*. A MV100 strain was constructed by integrating *kan*MX gene into the coding region of the yeast *MLH1*. By this intervention the ability of MMR to repair insertion/deletion loops on microsatellite GT tract was completely disrupted, with 79-fold greater mutation rate than in the wild-type (Figure 2).

For *in vivo *replacement of the yeast *MLH1 *gene by its human ortholog, we first integrated the CORE cassette into the yeast *MLH1 *gene. Experiments replacing the CORE cassette at codon 73 of the yeast *MLH1 *coding region with complete human *MLH1 *cDNA failed (data not shown). It was shown before in yeast, that MLH1 can play a role in regulating mitotic recombination [26] and we believe that its disruption, in our case by the integrated CORE cassette, could have a negative impact on recombination events needed for successful replacement of the cassette. For this reason we engineered an alternative gene fusion by integrating the CORE cassette after the yeast *MLH1 *stop codon, thus maintaining the integrity of the yeast gene. Replacement of this fusion construct with the complete human *MLH1 *cDNA was now feasible. However, human MLH1 alone did not complement the yeast ortholog and exhibited a 28-fold increase in mutation rate over the wild-type (Figure 2). Using the same strategy we then replaced the yeast *PMS1 *with the complete human *PMS2 *cDNA in the strain harbouring the *hMLH1 *gene. Co-expression of human MLH1 and PMS2 (strain MV400) indicated that the exogenous hMLH1-hPMS2 complex was able to fully restore the MMR function in yeast cells (Figure 2). Mutation rate was the same as observed in the wild-type strain (*P *= 0.89). However, the MV300 strain expressing *hPMS2 *alone exhibited a 40-fold higher mutation rate than that in the wild-type parental strain.

### The described system is operational for functional characterization of hMLH1 alterations

Our approach was shown to be operational for functional analysis of *hMLH1 *mutations by assessing eight different *hMLH1 *missense mutations found in cancer patients. Two variants (T117M and I219V) have already been evaluated by several different functional assays (Table 1). Because of the consistency of data obtained from the assays and their correlation with clinical reports, these variants were included as experimental controls in our study. We further analyzed the impact on phenotype of six missense mutations (A92P, S93G, K618R, K618T, Y646C and R659Q). An additional, non-cancer-associated frameshift mutation (1955 del 1 bp), which determines an early stop codon at position 660, was used to evaluate the impact of a clear loss-of-function mutation. All nucleotide rearrangements resulting in relevant codon changes were introduced *in vivo *into strain MV400 co-expressing *hMLH1 *and *hPMS2 *genes.

**Table 1:** Comparison of published functional and clinical data on hMLH1 variants

MLH1 alteration	Functional and biochemical assays		Clinical data				
		
	Assay type	Outcome^a^	MSI^b^	MLH1 IHC^c^	Age^d^	Family history/segregation	Ref.

A92P^e^							[30]

S93G	*In vivo *MMR assay [11,10]	- -			70	YES/	[9]
	*In vitro *MMR assay [11,9,3]	- - -	YES	POS	90	YES/	[32]
	*In vitro *protein interaction assay [9,3]	- -	YES	NEG	53	YES/	[32]
	MLH1 expression/localization [11,9]	- -					[33]
					65	YES/YES	[3]

T117M^e^	*In vivo *MMR assay [11,13]	+ +	YES				[34]
	*In vitro *MMR assay [11,12]	+ +			40	YES/	[35]
	*In vivo *protein interaction assay [7]	+	YES	NEG	43	YES/	[36]
	*In vitro *protein interaction assay [7,12]	± ±	NO	POS	42	YES/	[36]
	MLH1 expression/localization [11,7,13,16]	+ + + +	YES			YES/	[37]
			YES	POS	55	YES/	[38]
			YES	NEG			[39]

I219V	*In vivo *MMR assay [11,13]	- -		POS	55		[36]
	*In vitro *MMR assay [11,9,12]	- - -					
	*In vivo *protein interaction assay [7]	-					
	*In vitro *protein interaction assay [9,12]	- -					
	MLH1 expression/localization [11,9]	± -					

K618R^e^			YES	NEG			[40]
					< 50	NO/	[41]

K618T^e^	*In vivo *MMR assay [11,13]	+ +	NO	POS		YES/NO	[43]
	*In vitro *MMR assay [11,9]	± -	NO	POS		YES/NO	[44]
	*In vivo *protein interaction assay [7]	-			42	NO/	[45]
	*In vitro *protein interaction assay [7,42,9,12]	+ + - +					
	MLH1 expression/localization [11,7,13,9]	± ± ± ±					

Y646C	*In vitro *MMR assay [9]	-	YES	NEG	48	YES/	[46]
	*In vitro *protein interaction assay [9,6]	- +	YES	NEG^f^	36	YES/	[9,47]
	MLH1 expression/localization [9,6]	- -	YES	NEG	48	YES/	[6]
			YES	NEG	51	YES/	[6]


R659Q^e^	*In vivo *MMR assay [11]	±	YES	NEG	32	YES/	[9]
	*In vitro *MMR assay [11,9]	- -					
	*In vitro *protein interaction assay [9]	-					
	MLH1 expression/localization [11,9]	± -					

^a^The outcome of each assay is given: +, pathogenic; -, non-pathogenic; ±, inconclusive. Each symbol refers to the assay referenced in section: Assay type.

^b^Microsatellite instability.

^c^Immunohistochemical staining of MLH1: POS, positive; NEG, negative.

^d^Patients' age at cancer onset.

^e^The variant was also found in patients carrying a second MMR alteration. Those patients were excluded from the Table.

^f^Immunohistochemical staining of PMS2. In this case MLH1 staining was positive.

The results of functional characterization of the hMLH1 variants are presented in Table 2. The amino acid alteration A92P did not alter MMR function, since the mutation rate was the same as observed in the wild-type parental MV400 strain. The 1.8-fold greater mutation rate in the strain expressing K618T variant, in comparison with the parental MV400 strain, was also not significant (*P *= 0.69). Six of the *hMLH1 *codon changes analyzed encode proteins which were observed in this study to support intermediate efficiencies of DNA MMR. The mutation rates measured in strains expressing *hMLH1 *variants S93G, T117M, I219V, K618R, Y646C and R659Q were significantly lower than in the *mlh1 *deficient yeast strain MV100 (*P *< 0.0001). Mutation rates of T117M, Y646C and R659Q were also clearly (P < 0,0001) greater (12.3-, 15.8- and 7.9-fold, respectively) than those obtained in the strain MV400 co-expressing wild-type human orthologs. A frameshift mutation 1955 del 1 bp, an experimental control never reported in HNPCC-patients, exhibited complete loss of MMR activity as expected. The alteration conferred a 82.7-fold higher mutation rate, than exhibited by the wild-type parental MV400 strain.

**Table 2:** Functional characterization of hMLH1 variants in the *in vivo *yeast-based system

MLH1 alteration	Nucleotide Change	Mutation rate (95% CI)	Functional classification	Summary of other functional studies	Summary of clinical data
**Missense variant**					
A92P	274G>CA	2.07 × 10^-6^ (1.17 × 10^-6 ^- 2.96 × 10^-6^)	**non-pathogenic**		
S93G^b^	277A>G	6.22 × 10^-6^ (2.50 × 10^-6 ^- 9.94 × 10^-6^)	**probably** **non-pathogenic**	non-pathogenic	probably pathogenic
T117M	350C>T	2.76 × 10^-5^ (1.28 × 10^-5 ^- 4.23 × 10^-5^)	**pathogenic**	pathogenic	pathogenic
I219V	655A>G	6.50 × 10^-6^ (2.20 × 10^-6 ^- 1.08 × 10^-5^)	**probably** **non-pathogenic**	non-pathogenic	non-pathogenic
K618R^b^	1853A>G	4.99 × 10^-6^ (3.26 × 10^-6 ^- 6.72 × 10^-6^)	**probably** **non-pathogenic**		probably pathogenic
K618T^a^	1853A>C	3.97 × 10^-6^ (1.78 × 10^-6 ^- 6.16 × 10^-6^)	**non-pathogenic**	inconclusive	non-pathogenic
Y646C	1937A>G	3.54 × 10^-5^ (1.78 × 10^-5 ^- 5.31 × 10^-5^)	**pathogenic**	non-pathogenic	pathogenic
R659Q	1976G>A	1.77 × 10^-5^ (8.52 × 10^-6 ^- 2.70 × 10^-5^)	**pathogenic**	non-pathogenic	pathogenic
**Frameshift mutation**					
	1955 del 1 bp	1.86 × 10^-4^ (1.30 × 10^-4 ^- 2.43 × 10^-4^)	**loss-of-function**		

^a^This variant is functionally indistinguishable from the wild-type (*P *> 0.05).

^b^Functional significance of the variant did not correlate with available clinical data.

## Discussion

Hundreds of *hMLH1 *alterations have been found in HNPCC patients and for many, especially those leading to amino acid substitutions, the pathogenicity is still difficult to interpret. These non-truncating mutations should be functionally characterized. For this purpose, a number of yeast-based assays evaluating the functional significance of MMR variants have been developed, based on the fact that MMR has been conserved throughout eukaryotic evolution. Because of conservation of cellular functions from yeast to mammals and the ease of genetic manipulation in yeast *S cerevisiae*, this unicellular organism has been used before for assessing patogenicity of other cancer-related genes (eg. BRCA1 and p53) [27,28]. Besides many *in vitro *functional assays that require separate characterization of multiple functional properties [9,12], *in vivo *tests have been developed which appear to be more reliable [7,10,11,13]. However, it is important to note that, with known *in vivo *yeast assays, only mutations occurring in evolutionarily conserved regions in humans and yeast could be functionally analyzed, due to the lack of homology between human and yeast genes [14,15]. Moreover, clinical data associated with the disease usually lack to correlate with functional analysis data (for references see Table 1). Considering the fact that the efficiency of mismatch repair is highly affected by the degree of expression of the *MLH1 *gene [15] (variable in an episomal way), we have proposed a yeast-based *in vivo *system co-expressing chromosome-integrated human *MLH1 *and human *PMS2 *genes. With this intervention, the expressions of both human genes were controlled by the orthologous yeast promoters. Besides that, the expression of gene in chromosome seems to be region-depended. Higher-expressed and lower-expressed regions have been reported along yeasts' chromosome III. Moreover, the stability of foreign gene in chromosome is better than that in plasmids [29].

The MLH1 protein contains an amino-terminal ATPase domain and a carboxy-terminal domain. According to Ellison's conclusion [10] and to our unpublished data, the human ATP domain is functional in yeast. The human ortholog alone does not restore MMR activity in *mlh1*-deficient yeast cells when expressed episomally [13]. The same appeared to be true with the chromosomal integration of the entire *hMLH1 *performed in our study (Figure 2). However, additional replacement of yeast *PMS1 *with *hPMS2 *(which encodes a heterodimeric partner of hMLH1) directly on the yeast chromosome, in a strain expressing human MLH1 protein, resulted in complete restoration of MMR function (Figure 2).

Co-expression of human *MLH1 *and *PMS2 *proved to be effective for evaluating the pathogenicity of alterations found throughout the coding region of human *MLH1 *gene. Nine different *hMLH1 *alterations were shown to bring about from little or no effect on protein function to complete loss of MMR activity (Table 2). Before interpreting the pathogenicity of these *MLH1 *alterations, the appropriate tolerance level for functional proficiency had to be established. For this reason, hMLH1 missense alteration I219V was included in the assay as the functional control. This hMLH1 modification is a known harmless polymorphism consistently reported to have no functional effect on MLH1 (for reference see Table 1). In our test system the variant exhibited a significantly greater mutation rate than the wild-type. We therefore propose that 3-fold increase in mutation rate over the wild-type is the reasonable threshold between harmless variants on one side, and the variants with reduced MMR activity that may contribute to cancer susceptibility on the other side of a threshold. The proposed treshold might change, as the pathogenicities of more variants with proven phenotypes are assessed with the described approach.

Accordingly, the amino acid changes A92P, S93G, K618R and K618T were classified as non-pathogenic variations (Table 2). S93G is associated with a late age of onset, but has been reported in biochemical studies to have no functional effect on MLH1. The latter was confirmed in our study. The alteration K618T has been functionally characterized with various biological tools, with controversial results as to its pathogenicity. Our results correlate with clinical studies, in which K618T was reported not to co-segregate with the disease and in which affected carriers showed microsatellite stability and normal immunohistochemical staining of MLH1. (Table 1). Modifications A92P and K618R were functionally characterized for the first time. The novel amino acid change A92P was found in a patient with gastric carcinoma, and reported as probably pathogenic from its predicted structural changes [30]. The K618R modification was identified in colorectal cancer patients, however the clinical data are rather incomplete. The results of our assay suggest that these two modifications may not be causally associated with the observed clinical phenotype in mutation carriers (Table 1). Nevertheless, A92P, S93G, K618R and K618T must not be considered as absolutely non-pathogenic. Some alterations, despite being functionally proficient, exhibited reduction of protein efficiency that was significant compared to the wild-type, and mutation carriers may still, with low penetrance, express a particular phenotype. Therefore functional data have to be considered carefully alondside clinical studies that need to be undertaken with much more caution.

According to our assay, a further four alterations, including the functional control - a frameshift mutation 1995 del 1 bp - should be considered as pathogenic variants. As assumed before, the missense mutations did not completely destroy protein function and are thus expected to result in incomplete clinical penetrance [31]. In this study, we confirmed previous reports showing that the T117M variant may be associated with pathogenicity in HNPCC (Table 1). Functional characterization of another two variants (Y646C and R659Q) was, however, not consistent with other functional studies, in which they were identified as non-pathogenic (Table 2). However, carriers of these variants developed cancer at early-middle age and showed HNPCC clinical features (Table 1). The discrepancy between our assay and previous functional studies can be explained by the conclusion that subtle functional alterations may not be revealed with *in vitro *biological tools [9]. In our study, complete elimination of MMR function was only established with the frameshift mutation 1995 del 1 bp.

## Conclusion

We have shown, in contrast to *in vitro *studies, that co-employing the chromosome-integrated *hMLH1 *and *hPMS2 *genes in yeast not only enabled pathogenic *MLH1 *variants to be discriminated from polymorphisms that have no effect on MMR function, but also enabled variants resulting in reduced MMR activity to be identified and functionally classified. Moreover, the results correlated with clinical data in five out of seven *hMLH1 *variants. Our yeast-based *in vivo *system may thus be used for functional characterization of variants with incomplete clinical data, since precise quantification of *MLH1 *variants is critical for effective early diagnosis and prognosis, as well as for genetic counselling of affected individuals.

## Competing interests

The authors declare that they have no competing interests.

## Authors' contributions

MV participated in the design of the study, performed the statistical analysis and drafted the manuscript. MV and NZ carried out the experiments. AC and PH participated in the design of the study. RK coordinated the study and helped to draft the manuscript. All authors read and approved the final manuscript.

## Pre-publication history

The pre-publication history for this paper can be accessed here:

http://www.biomedcentral.com/1471-2407/9/382/prepub
